# Effects of dietary electrolyte balance and calcium supply on mineral and acid−base status of piglets fed a diversified diet

**DOI:** 10.1017/jns.2020.10

**Published:** 2020-05-29

**Authors:** M. Bournazel, M. J. Duclos, F. Lecompte, D. Guillou, C. Peyronnet, A. Quinsac, N. Même, A. Narcy

**Affiliations:** 1 INRAE, Université de Tours, UMR BOA, 37380 Nouzilly, France; 2 MiXscience, Campus du Groupe Avril, 2/4 avenue de Ker Lann, 35172 Bruz, France; 3 Plateforme CIRE, Service imagerie, UMR 0085 PRC, INRA centre val de Loire, 37380 Nouzilly, France; 4 Terres Univia, 11 rue Monceau, 75008 Paris, France; 5 Terres Inovia, 11 rue Monge, Parc industriel, 33600 Pessac, France

**Keywords:** Dietary electrolyte balance, Calcium, Acid−base balance, Bone

## Abstract

Dietary electrolyte balance (dEB) is known to affect acid−base status and mineral metabolism, but is rarely considered in diet formulation for pigs. Yet, the use of a wide variety of local feedstuffs in Europe contributes to lowering the dEB and increasing the fibre content. Hence, mineral requirements may be modified and skeletal health affected. Therefore, the effects of a lower dEB and a higher dietary Ca level on acid−base balance and mineral status were assessed in young pigs fed a diversified diet. A total of twenty-four weaned pigs were fed a control moderate-dEB diet (C) or a diversified moderate-dEB (D), low-dEB (D-A) or low-dEB supplemented with Ca (D-CA) diet. Growth performance, venous blood gas and chemistry, urine pH, mineral balance and femur characteristics were determined. With an equivalent dEB compared with the C diet, the D diet caused an acidification of the urine and increased the excretion of P as a result of a higher dietary content of S. Low-grade metabolic acidosis occurred in piglets fed the D-A diet with changes at systemic and urine levels. A higher excretion of ammonia and P in urine was observed and some bone characteristics tended to be negatively affected. Ca supplementation partially counteracted the effects of low-grade acidosis. Urine excretion of P and ammonia was alleviated and bone characteristics improved. In conclusion, a higher Ca supply must be considered in more diversified diets to counteract the risk of evolving towards low-grade metabolic acidosis which can negatively affect bone.

The maintenance of acid−base balance is crucial to ensure normal metabolic and enzymic processes. To maintain homeostasis, the ingestion/production of H^+^ must be balanced through an effective removal of these ions from the body^(1)^. In this regard, buffer systems are activated to cope with changes in blood pH and avoid acidaemia^(2)^. However, a prolonged dietary acid load can result in a state of low-grade metabolic acidosis characterised by minor changes in blood pH, within the range considered normal, but associated with several metabolic disturbances^(3)^. Among them, reduced Ca balance and increased bone resorption have been previously observed^(4,5)^. The skeleton is a reservoir of labile bases and cations which can be mobilised for the maintenance of blood pH and excretion of acid loads^(6)^. Consequently, acid−base disturbances may induce adverse effects on bone strength in the long run from an early stage onward^(7)^.

In pig nutrition, dietary electrolyte balance (dEB) is not widely used when formulating feed even though the maintenance of this parameter in pigs is crucial for growth^(8,9)^. Although the dEB is generally quite far from the threshold below which growth is reduced, it can lead to an overload of acid that needs to be eliminated from the body, thereby inducing mineral metabolism disorders^(10)^. In Europe, the trend to reduce the use of soyabean meal in favour of local raw materials as well as the use of free amino acids supports the need to take the dEB into account. Soyabean meal is characterised by high K content, a decrease of its use resulting in a significant reduction of the dEB of the diet^(11)^. These ingredients are also particularly rich in fibre which may interfere with the dEB^(12)^. In conjunction, the current trend in reducing the Ca content in pig diets (e.g. to optimise P digestibility and phytase efficiency) may intensify the deleterious effect of acidosis on the skeleton. According to Létourneau-Montminy *et al*.^(13)^, reducing the dietary Ca level in P-adequate diets improves P digestibility and growth performance, but negatively affects the overall retention of P due to the specific requirement of both minerals, i.e. Ca and P, for the development of bone tissue. In this condition of reduced Ca supply, the risk of evolving towards low-grade metabolic acidosis thus raises questions about the maintenance of skeletal integrity in pigs^(14)^. Therefore, the present experiment was designed to study the effects of a lower dEB and a higher dietary Ca level on the acid−base balance and mineral status of young pigs fed a diversified diet, i.e. containing various local feedstuffs.

## Materials and methods

The experimental protocol was approved by the Regional Ethics Committee on animal experimentation (Rennes, France) and was conducted under the guidelines of the French Ministry for Animal Research (Paris, France; authorisation no. 02402.03).

### Experimental diets

All treatments were iso-energetic, iso-proteineous and equivalent in digestible amino acids. They contained maize and soyabean meal and equivalent amounts of barley (26 %) and wheat (17⋅7 %; Table 1). The control diet (C) corresponded to a standard weaner diet without antibiotics, formulated to meet the requirements of 10 to 25 kg pigs. The second diet (D) was enriched in local feedstuffs and contained 8⋅0 % wheat bran, 6⋅0 % sugar beet pulp and 5⋅0 % wheat distillers' dried grains with solubles (DDGS) at the expense of maize and soyabean meal. The dEB was maintained at 148 mEq/kg like C using 4⋅8 % potassium citrate. The third diet (D-A) was formulated in the same way as D, but contained 3⋅35 % potassium chloride (KCl) and 9⋅40 % calcium chloride dihydrate (CaCl_2_.2H_2_O) in order to decrease the dEB to −24 mEq/kg. The fourth diet (D-CA) was similar to D-A, but 4⋅83 % calcium carbonate (CaCO_3_) was added to increase the level of Ca, without modifying the dEB. Digestible P was equivalent across treatments and the Ca level was adjusted to obtain a Ca:digestible P ratio of 2⋅6 in all diets^(15)^, except for the D-CA diet in which it was increased to 3⋅2.

**Table 1.** Composition of experimental diets (as-fed basis)

	C	D	D-A	D-CA
Ingredients (g/kg)*				
Maize	303	175	173	168
Soyabean meal	217	76⋅5	76⋅5	76⋅7
Barley	260	260	260	260
Wheat	177	177	177	177
Wheat bran	–	80⋅0	80⋅0	80⋅0
Sugar beet pulp	–	60⋅0	60⋅0	60⋅0
Wheat DDGS	–	50⋅0	50⋅0	50⋅0
Casein HCl	7⋅80	54⋅5	54⋅2	54⋅2
Soya oil	–	27⋅0	28⋅0	28⋅0
Calcium chloride (2H_2_O)	–	–	9⋅40	9⋅40
Monocalcium phosphate	10⋅2	11⋅2	11⋅2	11⋅2
Calcium carbonate	8⋅95	6⋅60	–	4⋅83
Potassium citrate		4⋅80	–	–
Salt	3⋅75	3⋅38	3⋅38	3⋅38
Potassium chloride	–	–	3⋅35	3⋅35
l-Lysine HCl	4⋅60	4⋅38	4⋅38	4⋅38
l-Threonine	1⋅65	2⋅14	2⋅13	2⋅13
dl-Methionine	1⋅56	1⋅88	1⋅88	1⋅88
l-Tryptophan	0⋅45	0⋅62	0⋅62	0⋅62
Chemical characteristics (g/kg)†				
DM	891	898	896	894
NE (MJ/kg)	9⋅66	9⋅68	9⋅67	9⋅62
Crude protein	174	174	173	173
Lipids	26⋅0	46⋅1	51⋅1	51⋅6
TDF	(110)	(137)	(176)	(165)
Insoluble fibre	(94⋅6)	(121)	(111)	(140)
Soluble fibre	(16)	(16)	(65)	(25)
Digestible P	3⋅0	3⋅0	3⋅0	3⋅0
Total P	5⋅7	5⋅6	6⋅5	6⋅2
Ca	7⋅7 (6⋅9)	7⋅6 (6⋅5)	7⋅7 (6⋅4)	9⋅5 (8⋅2)
Ca:dP	2⋅6	2⋅5	2⋅6	3⋅2
K	7⋅5 (7⋅2)	7⋅5 (7⋅3)	7⋅5 (7⋅9)	7⋅5 (7⋅8)
Na	1⋅6 (1⋅5)	1⋅6 (1⋅2)	1⋅6 (1⋅2)	1⋅6 (1⋅4)
Cl	4⋅0 (5⋅1)	4⋅0 (4⋅9)	10 (11)	10 (12)
dEB (mEq/kg)	148 (104)	148 (99)	−24 (−60)	−24 (−82)
S	1⋅9 (2⋅0)	1⋅9 (2⋅3)	1⋅9 (2⋅4)	1⋅9 (2⋅3)

C, control low-fibre diet; D, diet enriched in local feedstuffs with a normal dietary electrolyte balance; D-A, diet enriched in local feedstuffs with a low dietary electrolyte balance; D-CA, D-A diet supplemented with Ca; DDGS, distillers' dried grains with solubles; NE, net energy; TDF, total dietary fibre; dEB, dietary electrolyte balance.

* Premix (0⋅50%) was added in all diets: 10 000 IU (3000 μg) vitamin A (retinol); 2000 IU (50 μg) vitamin D_3_ (cholecalciferol); 20 mg vitamin E (tocopherol); 2 mg vitamin K_3_ (menadione); 2 mg vitamin B_1_ (thiamine); 5 mg vitamin B_2_ (riboflavin); 20 mg vitamin B_3_ (vitamin PP, niacin); 11 mg vitamin B_5_ (calcium pantothenate); 5 mg vitamin B_6_ (pyridoxine); 0⋅2 mg vitamin B_8_ (biotin, vitamin H); 1 mg vitamin B_9_ (folic acid); 0⋅03 mg vitamin B_12_ (cyanocobalamin); 600 mg choline chloride; 104 mg Fe (FeCO_3_); 20 mg Cu (CuSO_4_); 40 mg Mn (MnO); 99 mg Zn (ZnO); 1 mg Co (CoCO_3_); 0⋅6 mg iodine (Ca(IO_3_)_2_); 0⋅3 mg Se (Na_2_SeO_3_); 2⋅95 g Ca (CaCO_3_).

† Analysed values in parentheses.

### Animals and experimental procedures

The experiment included twenty-four castrated male Piétrain × (Landrace × Large White) piglets weighing 9⋅6 ± 0⋅5 kg at 28 d old (day 0 of the experiment). The animals were regularly monitored by qualified and authorised staff in order to immediately intervene in case of a problem. They were housed individually in cages (0⋅6 × 0⋅8 m) equipped with a grid and a slurry pit under a slatted floor for faeces and urine collection. They were fed *ad libitum* and had free access to water. The ambient temperature of the housing was maintained at 28°C and decreased to reach 24°C at the end of the experiment. At day 8, blocks of four pigs were created, each from the same litter and with a similar body weight, resulting in six blocks of male piglets. Piglets within a block were allocated to one of the four dietary treatments (six piglets per diet). The piglets were offered an equal quantity of feed daily that was distributed in two equivalent meals corresponding to 4 % of their body weight. Daily feed intake was recorded individually during the experimental period. The collection period started on day 14 and ended on day 22 after an overnight fast to ensure that the intestine was empty. Faeces were collected at meal times (twice per d) and pooled per pig. Finally, faeces were mixed thoroughly with water, sampled and weighed. The urine was acidified by adding 3 ml of 10 % sulfuric acid/l of urine to maintain a pH <2 (to prevent microbiota development) and was also collected individually and stored at −20°C. At day 25, four pigs per diet were fitted surgically with a catheter. After an overnight fast, the catheter was inserted into the jugular vein after injection of an appropriate anaesthetic for piglets (premedication with Imalgène 1000 (Merial); 1 ml per 10 kg of body weight by the intramuscular route, 5 to 10 min before surgery) under general anaesthesia (administrated by inhalation with a face mask of isoflurane; Elvetis) in a surgery room. The catheter tube was inserted under the skin, externalised at the dorsum of the neck, and connected to a bag taped to the skin. The piglets were then returned to their cages to wake up and rest. The catheters were flushed every 2 d with 5 ml of sterile saline solution containing 1 % heparin. After 10 d of adaptation (day 35) to the catheter and to handling by humans, blood samples were taken during 8 h. Blood parameters (pH, pCO_2_, HCO_3_^−^, anion gap, Na^+^, K^+^, Cl^−^) were immediately measured using a blood gas analyser (ABL80 Flex Basic; Radiometer S.A.S.). Simultaneously, urine was collected to measure the pH and ammonia excretion. The piglets were weighed at day 0, day 8 and day 36. At the end of the experiment, individual pigs received half of their daily feed allowance in the morning and the other half 2.5 h before slaughter. The piglets were stunned electrically and killed by exsanguination at the end of the experiment. The femurs were collected and stored at −20°C for further analyses.

### Analyses

All samples were analysed in duplicate. DM of the diets was determined after drying at 103°C for 4 h. Insoluble, soluble and total dietary fibre was quantified according to the method of Prosky *et al.*^(16)^. One sample of faeces per pig was analysed for DM content and one sample was freeze-dried. Freeze-dried faecal samples were ground through a 1 mm grid. Samples were then ashed at 550°C for 8 h in a muffle furnace and solubilised with 16 m-nitric acid and 30 % hydrogen peroxide on a digestion block to dryness and finally diluted in 0⋅4 m-nitric acid. Total P, Ca, K, Na, S and Cl were measured in the diets, faeces and urine to determine the apparent total tract digestibility (ATTD) and apparent retention (AR) and in the femurs to determine mineral contents. Total P, Ca, Mg, K, Na and S were analysed using an inductive coupled plasma atomic emission spectrometer (ICP OES Thermoscientific^TM^ iCAP^TM^ 7200; method 990.08; AOAC International, 2006). Chloride was analysed using a kit (kit Chlorures; Biolabo). The right femurs were broken and then defatted using diethyl ether, dried at 103°C for 18 h, and finally ashed in a muffle furnace at 600°C for 16 h. The left femurs were scanned using a clinical computed tomography machine (Somatom Définition AS128; Siemens) with scan parameters set at 140 kV tube voltage and 500 mAs current. The image acquisition mode was 32 cm × 512 pixels matrix size with a slice thickness of 0⋅4 mm and a resolution of 625 μm. The images were converted into Digital Imaging and Communication in Medicine (DICOM) format for analysis using the SINGO.VIA software. Bone mineral density was estimated and given in Hounsfield units (HU) by the scanner software and later converted into g/cm^3^ using a phantom (Electron Density Phantom Model 062M). Total bone was defined in intervals from 200 to 3000 HU according to Militist *et al*.^(17)^. Trabecular and cortical bones were defined in intervals from 200 to 800 HU, and 801 to 3000 HU, respectively, according to Sherlock *et al*.^(18)^.

### Calculations and statistical analysis

ATTD was calculated as follows (DM basis):

ATTD (%) = ((*X*_intake_ (g/d) – *X*_excreted in faeces_ (g/d))/(*X*_intake_ (g/d))) × 100.

AR was calculated as follows:

AR (%) = ((*X*_intake_ (g/d) – *X*_excreted in faeces_ (g/d) – *X*_excreted in urine_ (g/d)/(*X*_intake_ (g/d)) × 100.

Urinary elimination was calculated as follows:

Urinary elimination (%) = (*X*_excreted in urine_ (g/d)/*X*_absorbed_ (g/d)) × 100,

with *X* being the mineral considered.

The widths of the diaphysis and medullary cavity were measured at the middle of the diaphysis, and the cortical thickness and area were calculated as:

Cortical thickness (cm) = diaphysis width (cm) – medullary cavity width (cm).

Cortical area (cm^2^) = π × (diaphysis width (cm)^2^ – medullary cavity (cm)^2^).

Determination of the sample size was based on a power calculation using the estimated mean and standard deviation for the ATTD of P (26⋅2 (sd 5⋅5) %) and blood pH (7⋅4963 (sd 0⋅017)) observed in previous studies^(19,20)^. Consequently, we estimated that in order to detect a change in the ATTD of P of approximately nine points, at a significance level of 0⋅05, with a power of 80 % in a two-sided *t* test, six animals per group should be used for the digestive trial. For blood analyses, four animals per group are necessary to detect a change of blood pH of 0⋅03 units.

All data were analysed using the MIXED procedure from SAS (SAS Institute Inc.) as appropriate for a randomised complete block design after the normality of the variables had been checked. The block factor represented six consecutive cages and piglets were the experimental unit. The model included diet as a fixed effect and block as a random effect. Block was considered to be a random effect. Differences were considered to be significant at *P* < 0⋅05 and to be a trend at 0⋅05 ≤ *P* < 0⋅10. When a significant difference between means was observed, *post hoc* significance between treatment means was determined using the Bonferroni test. All values are presented as means and pooled standard errors of the mean.

## Results

No adverse effects to the piglets' welfare occurred during the experiment, and, consequently, no medication was used. Operated animals recovered well from the surgery and blood samplings were performed without any issues. The chemical characteristics analysed in the experimental diets were in accordance with the expected values (Table 1) and crude protein was equivalent in the four diets. The inclusion of CaCl_2_ increased the dietary Cl content and decreased the dEB as expected. The inclusion of Ca increased the dietary Ca content and consequently the Ca:P ratio, as expected. Diets were formulated to have the same content of Na and K. The analyses, presented in Table 1, showed few variations, mostly for Na (from 1⋅2 to 1⋅5 g/kg).

### Growth performance

The D, D-A and D-CA diets increased the final body weight of the piglets compared with C (+1⋅5 kg; *P* = 0⋅023). The D diet increased the average daily gain (ADG) (+17 %; *P* = 0⋅008) and decreased the feed conversion ratio (FCR) (−11 %; *P* = 0⋅007) compared with the C diet (Table 2). The D-A diet decreased the ADG (−11 %; *P* = 0⋅008) and increased FCR (+10 %; *P* = 0⋅007) compared with the D diet. Feed intake was unchanged among treatments.

**Table 2.** Average weight, feed intake and feed efficiency of piglets during the experimental period* (Mean values and pooled standard errors)

	C	D	D-A	D-CA	sem	*P*
Initial BW (kg)	11⋅2	11⋅1	11⋅4	11⋅3	0⋅08	0⋅31
Final BW (kg)	24⋅6^b^	26⋅9^a^	25⋅4^a^	25⋅9^a^	0⋅27	0⋅023
ADG (g/d)	612^b^	718^a^	640^b^	662^a,b^	12⋅2	0⋅008
ADFI (g/d)	786	825	804	799	8⋅98	0⋅41
FCR	1⋅31^a^	1⋅17^b^	1⋅29^a^	1⋅23^a,b^	0⋅02	0⋅007

C, control low-fibre diet; D, diet enriched in local feedstuffs with a normal dietary electrolyte balance; D-A, diet enriched in local feedstuffs with a low dietary electrolyte balance; D-CA, D-A diet supplemented with Ca; BW, body weight; ADG, average daily gain; ADFI, average daily feed intake; FCR, feed conversion ratio.

a,b Mean values within a row with unlike superscript letters were significantly different (*P* < 0⋅05).

* *n* 6.

### Blood gas and urinary pH

The diets did not affect blood pH or K^+^ and Ca concentration (Table 3). The D diet significantly reduced urine pH compared with the C diet (−8⋅2 %; *P* < 0⋅001). Chloride addition (D-A) further reduced the urine pH compared with the D diet (−10 %; *P* < 0⋅001). Blood pCO_2_, HCO_3_^−^ and base excess were significantly reduced in piglets fed the D-A diet compared with those fed the C diet (−5⋅8, −4⋅7 and −22, respectively). Blood Na^+^ and Cl^−^ were significantly increased in piglets fed the D-A diet compared with those fed the C diet (+2 %) and ammonia excretion enhanced by 5-fold. Ca addition (D-CA) did not counteract the effect of Cl overload on blood and urine parameters, but tended to improve them. The D-CA diet reduced phosphataemia compared with the D diet and pCO_2_ and urinary pH compared with the C diet. Blood HCO_3_^−^ and base excess were lower and anion gap and Cl^–^ higher in pigs fed the D-CA diet than in pigs fed the C and D diets. The D-CA diet induced an increase in ammonia excretion compared with the C diet.

**Table 3.** Effect of dietary treatments on venous blood gas, serum chemistry, urine pH and ammonia excretion of piglets* (Mean values and pooled standard errors)

	C	D	D-A	D-CA	sem	*P*
Blood						
pH	7⋅466	7⋅470	7⋅463	7⋅447	0⋅002	0⋅22
pCO_2_ (mmHg)	45⋅12^a^	44⋅81^a,b^	42⋅49^b^	43⋅41^b^	0⋅23	<0⋅001
HCO_3_^−^ (mmol/l)	30⋅88^a^	30⋅46^a,b^	29⋅43^b,c^	28⋅68^c^	0⋅18	<0⋅001
Anion gap (mmol/l)	15⋅8^b^	15⋅8^b^	16⋅2^a,b^	16⋅6^a^	0⋅11	0⋅007
BE (mmol/l)	7⋅74^a^	7⋅17^a,b^	6⋅03^b,c^	5⋅31^c^	0⋅19	<0⋅001
Na^+^ (mmol/l)	136⋅8^b^	138⋅8^a,b^	139⋅4^a^	138⋅0^a,b^	0⋅35	0⋅040
K^+^ (mmol/l)	4⋅52	4⋅52	4⋅58	4⋅41	0⋅03	0⋅13
Cl^–^ (mmol/l)	97⋅0^b^	97⋅4^b^	99⋅4^a^	98⋅6^a^	0⋅18	<0⋅001
Ca^2+^ (mg/l)	87⋅8	87⋅9	88⋅1	87⋅1	0⋅39	0⋅77
Phosphate (mg/l)	80⋅0^a,b^	82⋅9^a^	79⋅9^a,b^	76⋅6^b^	0⋅76	<0⋅001
Urine						
pH	7⋅325^a^	6⋅720^b^	6⋅032^c^	6⋅459^b,c^	0⋅09	<0⋅001
Ammonia (mg)	30⋅4^c^	47⋅8^b,c^	142^a^	103^a,b^	10⋅8	<0⋅001

C, control low-fibre diet; D, diet enriched in local feedstuffs with a normal dietary electrolyte balance; D-A, diet enriched in local feedstuffs with a low dietary electrolyte balance; D-CA, D-A diet supplemented with Ca; BE, base excess determined by blood gas analysis.

^a,b,c^ Mean values within a row with unlike superscript letters were significantly different (*P*<0⋅05).

* *n* 4; results for individual pigs reflect the average analysis of eight samples per animal.

### Mineral balance

The D-A diet increased P intake, followed by the D-CA and D diets (from 2⋅32 for C to 2⋅69 g/d for D-A; *P* < 0⋅001), and increased the amount of P absorbed compared with the C diet (Table 4; 1⋅68 *v*. 1⋅37 g/d; *P* = 0⋅001). The D diet increased the amount of urine P (four-fold; *P* < 0⋅001) and its percentage of urinary elimination (+3⋅48 points; *P* < 0⋅001) compared with the C diet. These parameters were also significantly increased in piglets fed the D-A diet compared with the D diet (+75 %, *P* < 0⋅001; and +3⋅42 points, *P* < 0⋅001, respectively). The D-CA diet decreased these two parameters compared with the D-A diet (−79 %, *P* < 0⋅001; and −6⋅07 points, *P* < 0⋅001, respectively).

**Table 4.** Influence of dietary treatments on mineral balance of piglets* (Mean values and pooled standard errors)

	C	D	D-A	D-CA	sem	*P*
P						
Intake (g/d)	2⋅32^d^	2⋅49^c^	2⋅69^a^	2⋅60^b^	0⋅03	<0⋅001
Excreted in faeces (g/d)	0⋅95	0⋅94	1⋅01	1⋅06	0⋅03	0⋅14
Absorbed (g/d)	1⋅37^c^	1⋅56^a,b^	1⋅68^a^	1⋅54^a,b^	0⋅03	0⋅001
ATTD (%)	59⋅2	62⋅5	62⋅4	59⋅1	0⋅94	0⋅23
Urinary excreted (g/d)	0⋅02^c^	0⋅08^b^	0⋅14^a^	0⋅03^b,c^	0⋅01	<0⋅001
Retained (g/d)	1⋅36	1⋅48	1⋅53	1⋅50	0⋅03	0⋅071
AR (%)	58⋅4	59⋅4	56⋅1	57⋅8	0⋅95	0⋅52
Urinary elimination (%)†	1⋅48^c^	4⋅96^b^	8⋅38^a^	2⋅34^b,c^	0⋅72	<0⋅001
Ca						
Intake (g/d)	2⋅78^b^	2⋅70^b^	2⋅68^b^	3⋅47^a^	0⋅07	<0⋅001
Excreted in faeces (g/d)	1⋅22^a,b^	1⋅22^a,b^	1⋅15^b^	1⋅50^a^	0⋅05	0⋅026
Absorbed (g/d)	1⋅56^b^	1⋅48^b^	1⋅54^b^	1⋅97^a^	0⋅06	<0⋅001
ATTD (%)	56⋅2	54⋅8	57⋅4	56⋅9	1⋅24	0⋅88
Urinary excreted (g/d)	0⋅26^a,b^	0⋅09^c^	0⋅17^b,c^	0⋅33^a^	0⋅03	<0⋅001
Retained (g/d)	1⋅26^b^	1⋅38^b^	1⋅37^b^	1⋅64^a^	0⋅04	<0⋅001
AR (%)	45⋅4	51⋅2	51⋅1	47⋅3	1⋅13	0⋅060
Urinary elimination (%)†	17⋅4^a^	6⋅4^b^	11⋅1^b^	16⋅5^a^	1⋅18	<0⋅001
Na						
Intake (g/d)	0⋅61^a^	0⋅52^b^	0⋅48^c^	0⋅60^a^	0⋅01	<0⋅001
Excreted in faeces (g/d)	0⋅08^a^	0⋅09^a^	0⋅05^b^	0⋅05^b^	0⋅01	0⋅045
Absorbed (g/d)	0⋅54^a^	0⋅43^b^	0⋅44^b^	0⋅56^a^	0⋅01	<0⋅001
ATTD (%)	87⋅5^a,b^	82⋅1^b^	90⋅2^a,b^	92⋅1^a^	1⋅32	0⋅029
Urinary excreted (g/d)	0⋅11	0⋅09	0⋅13	0⋅18	0⋅02	0⋅15
Retained (g/d)	0⋅43	0⋅34	0⋅30	0⋅37	0⋅02	0⋅13
AR (%)	69⋅9	64⋅8	62⋅9	61⋅8	3⋅09	0⋅84
Urinary elimination (%)†	20⋅3	20⋅9	31⋅0	33⋅3	3⋅23	0⋅39
K						
Intake (g/d)	2⋅90^c^	3⋅06^b^	3⋅31^a^	3⋅29^a^	0⋅04	<0⋅001
Excreted in faeces (g/d)	0⋅82^c^	0⋅92^b,c^	1⋅18^a^	1⋅08^a,b^	0⋅04	0⋅001
Absorbed (g/d)	2⋅08	2⋅14	2⋅13	2⋅22	0⋅03	0⋅47
ATTD (%)	71⋅5^a^	70⋅0^a,b^	64⋅4^b^	67⋅3^a,b^	0⋅96	0⋅042
Urinary excreted (g/d)	1⋅68^a,b^	1⋅43^b^	1⋅79^a^	1⋅83^a^	0⋅05	0⋅019
Retained (g/d)	0⋅39^a,b^	0⋅71^a^	0⋅34^b^	0⋅38^b^	0⋅05	0⋅025
AR (%)	13⋅6^a,b^	23⋅3^a^	10⋅3^b^	11⋅6^b^	1⋅64	0⋅011
Urinary elimination† (%)†	80⋅9^a,b^	67⋅1^b^	84⋅1^a^	82⋅7^a^	2⋅28	0⋅018
S						
Intake (g/d)	0⋅80^b^	0⋅98^a^	0⋅98^a^	0⋅99^a^	0⋅02	<0⋅001
Excreted in faeces (g/d)	0⋅18	0⋅20	0⋅22	0⋅22	0⋅01	0⋅059
Absorbed (g/d)	0⋅62^b^	0⋅78^a^	0⋅77^a^	0⋅76^a^	0⋅01	<0⋅001
ATTD (%)	76⋅9	79⋅8	78⋅1	77⋅6	0⋅51	0⋅25
Urinary excreted (g/d)	0⋅25^b^	0⋅33^a^	0⋅38^a^	0⋅36^a^	0⋅01	<0⋅001
Retained (g/d)	0⋅36	0⋅45	0⋅39	0⋅41	0⋅01	0⋅087
AR (%)	45⋅5	45⋅7	38⋅8	41⋅3	1⋅17	0⋅19
Urinary elimination (%)†	40⋅9	42⋅8	49⋅1	46⋅9	1⋅31	0⋅12
Cl						
Intake (g/d)	2⋅09^c^	2⋅06^c^	4⋅72^b^	5⋅03^a^	0⋅30	<0⋅001
Excreted in faeces (g/d)	0⋅14^c^	0⋅18^b,c^	0⋅27^a^	0⋅26^a,b^	0⋅02	0⋅002
Absorbed (g/d)	1⋅95^c^	1⋅88^c^	4⋅45^b^	4⋅77^a^	0⋅29	<0⋅001
ATTD (%)	93⋅4^a,b^	91⋅4^b^	94⋅3^a^	94⋅8^a^	0⋅40	0⋅004
Urinary excreted (g/d)	1⋅48^b^	1⋅33^b^	3⋅82^a^	3⋅78^a^	0⋅27	<0⋅001
Retained (g/d)	0⋅48^b^	0⋅55^b^	0⋅63^a^	0⋅98^a^	0⋅06	0⋅003
AR (%)	22⋅9^a,b^	26⋅9^a^	13⋅1^b^	19⋅6^a,b^	1⋅68	0⋅014
Urinary elimination (%)†	75⋅5^a,b^	70⋅6^b^	86⋅1^a^	79⋅4^a,b^	1⋅83	0⋅009

C, control low-fibre diet; D, diet enriched in local feedstuffs with a normal dietary electrolyte balance; D-A, diet enriched in local feedstuffs with a low dietary electrolyte balance; D-CA, D-A diet supplemented with Ca; ATTD, apparent total tract digestibility; AR, apparent retention.

^a,b,c,d^ Mean values within a row with unlike superscript letters were significantly different (*P*<0⋅05).

* *n* 6.

† Percentage of the amount absorbed, as described in the Materials and methods section.

The D-CA diet increased Ca intake, the amount absorbed and retained compared with the other diets (+28 %, *P* < 0⋅001; +32 %, *P* < 0⋅001; and + 22 %, *P* < 0⋅001, respectively). The D diet decreased the amount of Ca excreted in the urine (−65 %; *P* < 0⋅001) compared with the C diet. The D and D-A diets decreased urinary elimination of Ca compared with the C and D-CA diets (−8⋅21 points; *P* < 0⋅001). The D-CA diet increased the amount of Ca excreted in faeces compared with the D-A diet (+30 %; *P* = 0⋅026) and increased the amount excreted in the urine compared with the D and D-A diets (+178 %; *P* < 0⋅001).

The D diet decreased Na intake compared with the C and D-CA diets (−14 %; *P* < 0⋅001). The D and D-A diets decreased the amount of Na absorbed (−21 %; *P* < 0⋅001). The addition of Cl in the diversified diets (D-A and D-CA) decreased the amount of Na excreted in the faeces (−41 %; *P* = 0⋅045). The D-CA diet increased Na intake compared with the D and D-A diets (+20 %; *P* < 0⋅001) and increased the ATTD of Na compared with the D diet (+10 points; *P* = 0⋅029).

The D diet increased K intake compared with the C diet (+6 %; *P* < 0⋅001). The D-A diet increased K excreted in faeces compared with the D diet (+28 %) and the C diet (+44 %; *P* = 0⋅001). The D-A diet decreased the ATTD of K compared with the C diet (−7⋅1 points; *P* = 0⋅042). D-A and D-CA increased K intake (+8 %; *P* < 0⋅001), the amount excreted in the urine (+27 %; *P* = 0⋅019) and the urinary elimination (+16⋅3 points; *P* = 0⋅018). Conversely, Cl addition decreased the amount of K retained (−49 %; *P* = 0⋅025) and the AR of K (−12⋅4 points; *P* = 0⋅011). The D-CA diet increased the amount of K excreted in faeces compared with the C diet (+32 %; *P* = 0⋅001).

Concerning S, the diversified diets (D, D-A and D-CA) increased intake (+23 %; *P* < 0⋅001), the amount absorbed (+24 %, *P* < 0⋅001) and the amount excreted in the urine (+43 %; *P* < 0⋅001). The amount of S excreted in the faeces and the amount retained tended to be increased.

The D diet did not affect the Cl balance. On the contrary, D-A increased Cl intake (+127 %; *P* < 0⋅001), the amount of Cl excreted in the faeces (+69 %; *P* = 0⋅002) and the amount of Cl absorbed (+132 %; *P* < 0⋅001) compared with the C and D diets. The D-A diet decreased the AR of Cl (−19⋅8 points; *P* = 0⋅014) and increased urinary elimination (+15⋅5 points; *P* = 0⋅009) compared with the D diet. Chloride addition in the D diets (D-A and D-CA) increased the ATTD of Cl compared with the D diet (+3⋅2 points; *P* = 0⋅004), the amount of Cl excreted in urine (+170 %; *P* < 0⋅001) and the amount of Cl retained (+56 %; *P* = 0⋅003) compared with the C and D diets. The D-CA diet increased Cl intake (+7 %, *P* < 0⋅001) and the amount of Cl absorbed (+7 %; *P* < 0⋅001) compared with the D-A diet.

### Femur characteristics

Chloride addition in the diversified diets (D-A and D-CA) increased the width of the medullary cavity compared with the C diet (Table 5; +11 %; *P* = 0⋅014). The D-CA diet increased the diaphyseal width compared with the C diet (+13 %; *P* = 0⋅017) and the cortical area compared with the C and D-A diets (+29 %; *P* = 0⋅020). The D-CA and D diets increased P bone deposition compared with the C diet (+12 %; *P* = 0⋅045).

**Table 5.** Femur characteristics of piglets fed dietary treatments* (Mean values and pooled standard errors)

	C	D	D-A	D-CA	sem	*P*
Weight (g DM)	37⋅6	40⋅2	39⋅8	41⋅1	0⋅71	0⋅094
Ash (g)	15⋅7	16⋅8	16⋅3	16⋅9	0⋅21	0⋅081
Ash (% DM)	41⋅6	42⋅1	40⋅9	40⋅5	0⋅81	0⋅47
Morphometry						
Length (cm)	12⋅14	12⋅36	12⋅19	12⋅34	0⋅07	0⋅66
Diaphysis width (cm)	1⋅48^b^	1⋅59^a,b^	1⋅56^a,b^	1⋅67^a^	0⋅02	0⋅017
Medullar cavity width (cm)	1⋅07^b^	1⋅13^a,b^	1⋅17^a^	1⋅20^a^	0⋅02	0⋅014
Cortical thickness (cm)	0⋅42	0⋅46	0⋅39	0⋅47	0⋅02	0⋅22
Cortical area (cm^2^)	3⋅33^b^	4⋅09^a,b^	3⋅47^b^	4⋅44^a^	0⋅15	0⋅020
Bone mineral density (g/cm^3^)						
Entire bone	1⋅266	1⋅270	1⋅268	1⋅270	0⋅001	0⋅64
Trabecular	1⋅212	1⋅214	1⋅212	1⋅213	0⋅001	0⋅81
Cortical	1⋅677	1⋅670	1⋅680	1⋅666	0⋅003	0⋅29
Mineral content (g/femur)						
P	2⋅885^b^	3⋅224^a^	3⋅135^a,b^	3⋅213^a^	0⋅050	0⋅045
Ca	5⋅314	5⋅901	5⋅732	5⋅842	0⋅090	0⋅067
Na	0⋅226	0⋅243	0⋅243	0⋅239	0⋅004	0⋅18
K	0⋅115	0⋅116	0⋅117	0⋅111	0⋅003	0⋅89
S	0⋅083	0⋅087	0⋅097	0⋅087	0⋅004	0⋅30
Cl	0⋅119	0⋅129	0⋅126	0⋅126	0⋅002	0⋅27
Ca:P ratio	1⋅845	1⋅832	1⋅828	1⋅818	0⋅009	0⋅16

C, control low-fibre diet; D, diet enriched in local feedstuffs with a normal dietary electrolyte balance; D-A, diet enriched in local feedstuffs with a low dietary electrolyte balance; D-CA, D-A diet supplemented with Ca.

^a,b^ Mean values within a row with unlike superscript letters were significantly different (*P*<0⋅05).

* *n* 6.

## Discussion

With the same dEB as the C diet, the D diet had an acidifying effect at the urinary level. Higher total S content was observed in the D diet, probably due to the inclusion of DDGS. According to Kerr & Surshon^(21)^, the use of sulfuric acid in the dry grind process of DDGS leads to increased total S by 5-fold compared with maize. This results in higher urinary excretion, which partly explains the significant decrease in the urinary pH. Additionally, even if fermentations in the caecum were not enhanced in the diversified diets (data not shown), the production of organic anions other than SCFA, such as succinate, lactate and galacturonate^(22)^, or active fermentation in the distal part of the colon cannot be excluded^(23)^. The effect of these organic anions on acid−base balance is still not fully understood, but one of the hypotheses involves an anionic exchange between SCFA and HCO_3_^−^, transferred from the extracellular body fluids into the colonic lumen^(24)^. The diets were expected to be equivalent in terms of digestible P, but the increase in total P intake resulted in higher absorbed P. Despite the acidification of the urine in piglets fed the D diet compared with those fed the C diet, bone mineralisation was improved, probably due to a higher amount of absorbed P. However, the optimal level of bone mineralisation was probably not achieved due to a lack of Ca as suggested by the higher P and lower Ca urinary excretion, which illustrate a metabolic imbalance. Indeed, P and Ca are deposited together in the mineral matrix of bones in the form of hydroxyapatite^(25)^. Bone mineralisation thus depends on the availability of both minerals at the metabolic level, the lack of one of the two minerals inducing the excretion of the other^(26)^. Mineral digestibility was not affected by the higher content in dietary fibre of the diversified diets, which was mostly insoluble. Conversely, Dersjant-Li *et al*.^(12)^ showed that increasing dietary NSP content increased apparent digestibility of Cl, Na and K in the first part of the small intestine. Levrat-Verny *et al*.^(27)^ reported an improvement in Ca, Mg and Fe absorption in rats fed whole-wheat flour compared with those fed white flour, which was probably due to the microbial breakdown of fibre. Indeed, fermentation can lower hindgut pH and increase SCFA production, thus favouring solubilisation and absorption of minerals^(28–31)^. In the present study, we observed neither a decrease in caecal or colic pH, nor a modification of the SCFA profile (data not shown). The duration of the experiment or the gap in the fibre content between the C and D diets may have been insufficient to induce any significant effects.

Decreasing the dEB from 100 to −60 induces a deterioration of ADG and FCR without modifying feed intake. It could be thus hypothesised that the lower feed efficiency observed with the low dEB is related to higher energy costs for the maintenance of acid−base homeostasis. According to Patience & Wolynetz^(32)^, growth and feed intake appeared to be maximal for a dEB of 0 to 341 mEq/kg, but were decreased at −85 mEq/kg. From the results of Dersjant-Li *et al*.^(33)^, a restricted feeding level dEB ranging from −135 to 145 mEq/kg has little effect on the energy metabolism of young pigs, suggesting that the poor performance observed would be mainly related to a reduced feed intake. Changes at the systemic and urinary levels confirm that regulation processes occurred. The kidney contributes to the excretion of the acid load, allowing the plasma pH to be maintained in a physiological range. Part of the overload of dietary Cl was excreted in the urine with a counterbalance of cations, mainly in the form of NH_4_^+^ and, to a lesser extent, K^+^, Ca^2+^ and Na^+^. In response to metabolic acidosis, higher urinary ammonia excretion is the primary component of the increase in net acid excretion^(34)^. A six-fold increase in urine ammonia flux was also observed when reducing the dEB from 163 to −20 mEq/kg^(4)^. Indeed, renal gluconeogenesis and ammoniagenesis are stimulated to regenerate HCO_3_^−^ and provide NH_4_^+^ in equivalent amounts^(35)^. Then HCO_3_^−^ is returned to the systemic circulation, whereas ammonia (NH_4_^+^ and NH_3_) is excreted in the urine to regulate net acid excretion^(34)^. Glutamine is the primary metabolic source for ammoniagenesis and its renal uptake increases substantially during metabolic acidosis. The acidification also induced a higher loss of K from the body. Potassium is the main intracellular cation, primarily stored in muscle, and its concentration is finely maintained by Na-K pumps. This tight regulation is critical for normal cell function, and low-grade metabolic acidosis is known to induce a net loss of cellular K^(36)^. The difficulty in maintaining the intracellular pool of K could partly explain the observed loss of performance.

The acidification also resulted in a marked excretion of phosphate, which is the major H^+^ buffer system in urine. Its excretion is increased during acidosis as a result of a decrease of the preferentially transported form (HPO_4_^2−^), together with a direct effect of the luminal pH on the apical phosphate carriers (NaPiIIa and IIc) in the proximal tubule^(37)^. The additional urinary P output (3 g estimated on 30 d) represented the P content of the femur of a 2-month-old piglet. Although not significant, the acidification tended to depress some femur characteristics, suggesting a change in bone resorption or a decrease in bone mineral deposition. In agreement with Budde & Crenshaw^(20)^, a higher Cl retention was observed, which may suggest the storage of Cl in bone. This may contribute to the buffering of the acid load through the exchange of Cl for other anions such as phosphate, which in turn is released. However, the level of Cl in the femur did not change significantly, contradicting the hypothesis that bone was a storage compartment for excess Cl. This cannot be confirmed as only the femur was analysed, which is not representative of the overall skeleton. The femur is made up of cortical bone, and it is less vascularised compared with trabecular/spongy bone^(38)^. Consequently, the dynamics of exchanges with the blood could be different between those two types. The role of the skeleton could be less important than previously stated, especially since competition between the need to eliminate acid loads and the need to form bone probably occurs due to the high growth rate of piglets. According to Budde & Crenshaw^(20)^, feeding piglets for 3 weeks post-weaning with a lower dEB (−35 *v*. 212) did not impair bone mineral and ash content or bone breaking strength. This discrepancy between these results and those of the present study could be explained by the higher level of Ca used by the authors (9⋅6 *v*. 6⋅5 g/kg).

Currently, dietary Ca tends to be reduced as it can antagonise P absorption^(39)^ and deteriorate growth performance. As quantified by Létourneau-Montminy *et al*.^(13)^, increasing Ca from 5 to 8 g/kg induces a reduction of 0⋅10 g digestible P/kg regardless of the dietary P concentration. In the present study, increasing the Ca level from 6⋅4 to 8⋅2 g/kg was not detrimental to P digestibility. In addition, Ca supplementation tended to reduce the negative effect of Cl overload, but without fully counteracting its acidifying effect at systemic and urinary levels. Ca partly contributes to the neutralisation of Cl anions in the urine, with a consecutive sparing effect of phosphate, which is more easily reabsorbed. Additionally, the higher Ca supply may favour the deposition of P in the skeleton, thereby improving bone characteristics, particularly in the cortical area. As the cortical area represents the strongest part of the bone, higher mineral quality and strength can be expected, which could be determinant later during the growing period. It should be noted that bone mineral density was not affected. This is in agreement with Viguet-Carrin *et al*.^(40)^, who observed a significant increase in bone strength and femur length which was not accompanied by an increase in bone mineral density or bone markers in growing rats fed diets with high Ca levels. This result highlights the importance of Ca in the regulation of the acid−base balance beyond the components of the dEB.

### Conclusion

The results of the present study suggest that the increasing use of various local feedstuffs in feed formula requires adapting the dEB when formulating diets to avoid any over-excretion of P and ammonia. In parallel, a higher Ca level must be considered to counteract the negative effects of low-grade metabolic acidosis on P excretion and prevent bone alteration.
